# Hereditary profiles of disorderly transcription?

**DOI:** 10.1186/1745-6150-1-9

**Published:** 2006-04-02

**Authors:** Johannes WIM Simons

**Affiliations:** 1 Department of Toxicogenetics, MGC, Leiden University Medical Center, PO Box 9600, 2300 RC Leiden, The Netherlands

## Abstract

**Background:**

Microscopic examination of living cells often reveals that cells from some cell strains appear to be in a permanent state of disarray without obvious reason. In all probability such a disorderly state affects cell functioning.

The aim of this study was to establish whether a disorderly state could occur that adversely affects gene expression profiles and whether such a state might have biomedical consequences. To this end, the expression profiles of the 14 genes of the proteasome derived from the GEO SAGE database were utilized as a model system.

**Results:**

By adopting the overall expression profile as the standard for normal expression, deviation in transcription was frequently observed. Each deviating tissue exhibited its own characteristic profile of over-expressed and under-expressed genes. Moreover such a specific deviating profile appeared to be epigenetic in origin and could be stably transmitted to a clonal derivative e.g. from a precancerous normal tissue to its tumor. A significantly greater degree of deviation was observed in the expression profiles from the tumor tissues.

The changes in the expression of different genes display a network of interdependencies. Therefore our hypothesis is that deviating profiles reflect disorder in the localization of genes within the nucleus

The underlying cause(s) for these disorderly states remain obscure; it could be noise and/or deterministic chaos. Presence of mutational damage does not appear to be predominantly involved.

**Conclusion:**

As disturbances in expression profiles frequently occur and have biomedical consequences, its determination could prove of value in several fields of biomedical research.

**Reviewers:**

This article was reviewed by Trey Ideker, Itai Yanai and Stephan Beck

## Open peer review

Reviewed by Trey Ideker, Itai Yanai and Stephan Beck. For the full reviews, please go to the Reviewers' comments section.

## Background

Within a living cell there will always be "spontaneous" variation in functioning. The origin of this variation could be presence of mutational damage, random fluctuations (also known as noise) or deterministic chaos. Noise has been shown to affect cell functioning [1] and consequently it can be assumed that some degree of disorder will always be present in a cell. In theory, since extensive disorder could affect the health of the cell and thus ultimately the health of the individual, it would seem prudent to investigate whether a phenomenon like cellular disorder can be demonstrated and analyzed. Databases on gene transcription are now available that facilitate such an investigation.

In this study the word "disorder" is used as an inclusive term to describe "excessive variation in transcription irrespective of cause", thus random variation, deterministic chaos or presence of mutational damage could all be causally involved.

The questions we want to address are: 1) can excessive variation in transcription be demonstrated, 2) does such excessive variation have a degree of permanence and 3) does it play any role in health and disease.

That disorder in gene expression does occur and could be of relevance for understanding carcinogenesis is suggested by the following observations. Firstly, exposure of cells to carcinogens may lead to a state of "delayed, persistent genomic instability" [2] that can affect each aspect of cell structure and function and predisposes the cell to immortalization. Such genomic instability can also be transmitted to neighboring cells via the medium (bystander effect) [3,4] and thus does not depend on the presence of mutational damage within the cell. Persistent disorder in transcription might be implied from this unstable state.

Secondly, it is generally accepted that although mutations in oncogenes and/or tumor suppressor genes predispose cells to carcinogenesis, epigenetic and non-genetic mechanisms also play a role [5]. These processes ultimately lead to the cell acquiring a transformed phenotype with concomitant alterations in the life span or eventual immortalization [6]. Although telomerase is known to be expressed during this process of cell transformation, the way in which this gene becomes activated is still unknown [7,8]. Since the process of transformation appears to involve progressive deregulation of cell functioning, increasing disorder in transcription profiles may play a role.

Thirdly, since predisposition to tumorigenesis can be related to only a small change in the expression level of a single gene [9], even minor fluctuations in gene expression could have major consequences. Moreover it has recently been shown that noise in gene expression is biologically relevant as it is detrimental to organismal fitness [10].

Thus perturbations in the transcriptome could be present in cells that are in the process of malignant transformation and such disorder in the transcriptome might, in itself, be a driving factor in carcinogenesis.

The availability of the Cancer Genome Anatomy Project's (CGAP) SAGE (Serial Analysis of Gene Expression) database of human gene expression levels in a wide variety of cells [11] has enabled us to check our hypothesis and to establish that excessive variation in transcription can be constitutive and hereditary. These findings could prove to be of importance in various fields of biomedical research.

## Results and discussion

### The proteasome as a model system to investigate disorder in transcription

Although the ultimate aim of this investigation is to establish the existence of a state of cellular disorder that affects all transcription profiles, the first step involves the choice of only one expression profile that could serve as a model for all profiles in a complex system although it is evident that one expression profile will not represent the whole human transcriptome. The transcription profile of the genes that code for a cellular organelle, the 20S proteasome, was chosen to fulfill this role. Since a cellular organelle has a well-defined structure, a prerequisite for its assembly would be that the products of the genes involved be available in, ideally, the correct amounts. Therefore we assume that an optimal expression pattern for the transcription of the genes in question exists although, of course, it might turn out that the expression pattern can be influenced by factors like tissue type or response to stimuli. Sampling errors will affect the number of transcripts of the proteosomal genes found in a library. However, If the degree of variation turns out to be greater than that expected due to sampling, then this could be indicative of the existence of transcriptional disorder.

The 20S proteasome, a structure 15 nm in length with a diameter of 11–12 nm, is organized as four stacked rings with a central channel. This architecture is highly conserved from bacteria to man. Each rings consists of 7 different subunits, each located at a defined position [12], Alpha-type subunits and beta-type subunits form the two outer and two inner rings respectively. The 14 genes coding for these subunits are all regulated independently from each other and are located on different chromosomes. Thus 14 gene products are needed in equal amounts to build the proteasome. For our calculations we assume the existence of a preferential expression profile for these 14 genes and deviation from this preferential expression profile could indicate "disorder in transcription".

The tags (listed in Table 1) used to establish the degrees of expression of the proteasome genes were derived from the NCBI website [13] Unique cDNA tags are available for 6 of the 14 genes, whilst the remaining tags also detect expression of genes other than the proteasome genes. Therefore, additional tags specific for the expression of these non-proteasome genes were utilized to check their expression. However, in the available libraries, the expression of these non-proteasome genes appeared so rare that the counts obtained with the proteasome tags of Table 1 are reliable.

**Table 1:** List of tags used to identify the degree of expression of the proteasome genes.

Symbol	tag	UID	gene description	control tag
PSMA1	GTCTGCGTGC	Hs.82159	proteasome subunit, alpha type, 1	
		Hs.169942	ESTs	TCTAAGAGAA
PSMA2	GTTTAAATCG	Hs.181309	proteasome subunit, alpha type, 2	
		Hs.1290	complement component 9	TGTCCAAGGG
PSMA3	AAATTGTTCC	Hs.346918	proteasome subunit, alpha type, 3	
		Hs.270791	FLJ11437 fis	TGTAAATGAA
		Hs.60293	FLJ10883	TTTTGCCTGA
PSMA4	GACGTCTTAA	Hs.251531	proteasome subunit, alpha type, 4	
PSMA5	TTCACAAAGG	Hs.76913	proteasome subunit, alpha type, 5	
		Hs.11223	isocitrate dehydrogenase	ACCAAGGACT
PSMA6	GAGGTCCCTG	Hs.74077	proteasome subunit, alpha type, 6	
		Hs.121516	Rho GDP dissociation inhibitor	TGCCCAAGAG
PSMA7	AGGCGAGATC	Hs.233952	proteasome subunit, alpha type, 7	
		Hs.195464	filamin A, alpha	AGGCCGAGAT
PSMB1	CGGCTGGTGA	Hs.75748	proteasome subunit, beta type, 1	
		Hs.334775	MGC20255	AGAAAGTGGC
PSMB2	TCCTCCCTCC	Hs.1390	proteasome subunit, beta type, 2	
PSMB3	GGAGTCATTG	Hs.82793	proteasome subunit, beta type, 3	
PSMB4	AAGGAATCGG	Hs.89545	proteasome subunit, beta type, 4	
PSMB5	AGAAGTATAG	Hs.78596	proteasome subunit, beta type, 5	
PSMB6	GAGCGGGATG	Hs.77060	proteasome subunit, beta type, 6	
PSMB7	TGGCTAGTGT	Hs.118065	proteasome subunit, beta type, 7	
		Hs.283429	Smcx homolog	GGCGGTGTGT

In order to obtain data suitable for statistical analysis, only those libraries that have a total tag count of at least 24 for the expression of all the proteasome genes together were used. Additionally, only libraries derived from biopsies were used in order to circumvent any possible effects associated with tissue culture conditions. At the beginning of this study 60 libraries were available that met these criteria, 30 of these were derived from normal tissues and 30 from cancer tissues. A possible disadvantage of using these datasets is that they are derived from very different tissues. However, this was unavoidable due to the limited number of available libraries. Nevertheless, no significant difference between normal and tumor tissues existed for the total tag count per library (Wilcoxon: P = 0,274) or for the counted number of proteasome tags per library (Wilcoxon: P = 0,504). Therefore the groups are homogeneous with respect to these characteristics.

### Excessive variation in transcription of proteasomal genes in libraries derived from normal and tumor tissues

Expected frequencies of tags for the 14 genes of a library were obtained with the overall expression profile. This expression profile was derived from the sums of the tag counts in all 60 libraries (Table 2 A). The observed tag counts are shown in Table 3. The expected and observed tag counts were compared by chi-square. Of the 30 normal tissues, 13 deviated (P < 0,05) from the expected values, compared to 23 of the 30 tumor tissues. As the prerequisites for the application of the chi-square test were not met the probabilities of the chi-squares of the two groups were compared using Wilcoxon's test. The two groups proved to be significantly different from each other (P = 1.73 × 10^-6^), with tumor tissues being more disorderly. In conclusion it is apparent that excessive variation in transcription does occur and that as a group, tumor tissues show a significantly greater degree of variation in transcription than do normal tissues. However, the data also indicates that some normal tissues show excessive variation whilst some of the tumor tissues might still have a normal expression profile.

**Table 2:** Mean expression profiles of the proteasomal genes used for the calculation of the expected frequencies of tags.

	A		B		C		D		E		F	
	60 libraries		80 libraries		All 140 libraries		30 breast libraries		37 most disorderly		37 most orderly	
genes	tags	rel. freq.	tags	rel. freq.	tags	rel. freq.	tags	re. freq.	tags	rel. freq.	tags	rel. freq.
PSMA1	204	0,044	372	0,047	576	0,046	104	0,046	216	0,049	118	0,038
PSMA2	397	0,086	800	0,100	1197	0,095	188	0,084	406	0,091	290	0,093
PSMA3	81	0,018	191	0,024	272	0,022	40	0,018	73	0,016	78	0,025
PSMA4	321	0,070	517	0,065	838	0,067	160	0,071	265	0,060	263	0,084
PSMA5	169	0,037	263	0,033	432	0,034	44	0,020	143	0,032	109	0,035
PSMA6	578	0,125	1159	0,146	1737	0,138	327	0,146	574	0,129	449	0,144
PSMA7	335	0,073	499	0,063	834	0,066	189	0,084	323	0,073	210	0,067
PSMB1	410	0,089	729	0,092	1139	0,091	237	0,106	391	0,088	271	0,087
PSMB2	185	0,040	357	0,045	542	0,043	87	0,039	170	0,038	115	0,037
PSMB3	652	0,141	761	0,096	1413	0,112	409	0,182	523	0,118	326	0,104
PSMB4	197	0,043	230	0,029	427	0,034	67	0,030	175	0,039	99	0,032
PSMB5	121	0,026	219	0,028	340	0,027	25	0,011	128	0,029	67	0,021
PSMB6	370	0,080	594	0,075	964	0,077	141	0,063	344	0,077	230	0,074
PSMB7	589	0,128	1270	0,160	1859	0,148	224	0,100	708	0,159	468	0,150
	4609	1.000	7961	1.000	12570	1.000	2242	1,000	4439	1,000	3125	1,000

**Table 3:** Expression of the proteasome genes in the selected SAGE libraries.

																			deviation index	
GEO	total tags	tissue	PSMA1	PSMA2	PSMA3	PSMA4	PSMA5	PSMA6	PSMA7	PSMB1	PSMB2	PSMB3	PSMB4	PSMB5	PSMB6	PSMB7	Sum		log ratio	z-score
30 normal libraries																				
676	94876	brain	6	4	0	0	6	3	6	5	7	1	3	1	4	8	54		0,377	1,698
677	37642	breast	4	6	2	4	3	14	7	12	1	7	4	0	3	6	73		0,224	1,087
780	63227	breast	2	4	2	4	4	4	9	4	2	6	1	1	2	10	55		0,221	1,083
781	58444	breast	1	10	0	0	0	3	8	1	4	4	1	2	6	5	45		0,326	1,556
685	66483	prostate	8	15	2	8	6	11	20	19	4	28	29	9	9	21	189		0,274	2,856
688	28950	breast	1	3	3	8	1	9	5	4	2	7	1	0	4	6	54		0,232	1,037
691	7165	breast	1	5	1	3	1	7	1	1	0	4	1	0	1	2	28		0,235	0,911
692	12142	breast	1	7	0	2	0	2	0	2	1	3	0	0	2	4	24		0,181	1,063
695	58826	brain	2	2	1	1	5	5	1	4	0	2	0	1	5	7	36		0,270	1,244
708	41857	kidney	2	2	3	4	1	6	7	1	0	4	2	1	4	5	42		0,280	1,156
713	48548	brain	1	0	0	1	1	5	0	4	1	4	0	2	5	5	29		0,243	1,017
574	102359	retina	4	13	2	19	0	17	7	5	0	16	5	2	8	36	134		0,305	1,784
719	48552	ovary	6	14	0	10	3	7	4	13	9	9	8	2	7	8	100		0,230	1,453
572	59661	retina	3	1	1	5	2	6	6	6	2	8	2	0	9	16	67		0,256	1,081
573	105312	retina	2	9	4	14	3	8	5	5	3	14	5	1	8	29	110		0,243	1,481
760	49281	ovary	11	8	1	8	2	27	10	21	3	3	6	0	10	8	118		0,310	1,849
728	50179	colon	5	1	2	1	4	1	6	11	2	0	1	1	9	2	46		0,468	1,918
729	49593	colon	5	0	1	2	1	4	5	7	3	7	1	0	6	6	48		0,265	1,130
761	51280	brain	4	1	2	4	0	5	4	1	2	1	0	1	6	6	37		0,337	1,204
1499	84357	heart	6	16	4	7	7	28	11	14	1	17	1	5	14	27	158		0,302	1,005
819	53853	muscle	2	6	0	3	1	9	10	2	3	2	5	3	6	7	59		0,279	1,348
824	53875	muscle	5	3	2	2	3	13	9	3	2	4	0	6	10	9	71		0,291	1,492
785	66861	liver	2	14	2	9	12	14	2	14	3	15	2	4	7	27	127		0,273	1,504
762	89143	lung	1	15	6	21	7	27	2	10	6	22	1	4	10	23	155		0,369	1,597
786	77986	brain	3	4	0	4	1	8	1	3	2	4	0	5	4	24	63		0,284	1,744
763	63208	brain	4	0	0	5	0	2	4	5	3	7	4	0	5	5	44		0,292	1,284
3242	37292	skin	0	5	0	6	1	5	8	3	1	4	0	0	3	4	40		0,210	1,289
2386	55422	spinal cord	2	7	3	3	1	6	3	8	2	9	2	2	5	9	62		0,170	0,698
738	54096	peritoneum	2	1	0	0	1	2	5	4	3	4	1	0	4	4	31		0,253	1,051
739	59553	prostate	6	7	3	8	7	8	0	6	3	11	0	8	9	10	86		0,328	1,611
30 tumor libraries																				
2451	38634	brain	0	3	0	3	2	3	3	4	1	4	2	3	5	7	40		0,199	0,872
2443	80265	brain	2	15	0	10	2	15	8	10	7	13	1	3	8	12	106		0,229	0,981
2578	69513	brain	0	10	3	0	6	12	1	9	6	9	4	3	5	11	79		0,313	1,246
699	28159	brain	1	3	1	0	3	7	13	2	4	1	12	2	10	9	68		0,441	2,451
698	77004	brain	4	5	1	3	2	10	7	5	1	3	6	4	11	2	64		0,323	1,504
697	52479	brain	0	4	2	2	2	4	6	4	0	4	1	0	0	5	34		0,230	1,115
672	67386	breast	2	9	3	9	5	15	9	9	3	62	1	1	8	6	142		0,327	3,481
687	41378	breast	2	9	1	1	1	25	1	15	0	1	0	0	3	5	64		0,375	2,245
670	40223	breast	3	3	0	14	1	12	6	4	4	65	1	1	2	8	124		0,375	4,170
755	57686	colon	5	3	2	2	2	4	3	4	0	3	4	0	2	3	37		0,252	1,216
756	49064	colon	7	6	1	1	5	5	2	7	5	9	0	0	2	6	56		0,300	1,423
792	34537	brain	2	4	2	3	2	9	0	15	2	5	0	0	3	5	52		0,239	1,565
1497	46928	brain	6	8	0	2	5	6	0	4	9	9	0	5	6	9	69		0,331	1,651
793	56871	brain	9	9	3	8	3	13	2	17	0	7	2	0	4	13	90		0,281	1,471
696	70087	brain	3	11	0	3	4	5	11	13	11	10	6	3	15	13	108		0,257	1,561
765	61886	brain	4	3	0	2	2	5	13	9	8	10	0	0	11	10	77		0,291	1,714
745	60069	brain	2	6	0	7	1	29	6	3	0	5	0	0	12	15	86		0,295	1,908
1516	76168	brain	6	18	1	14	2	20	4	11	12	11	1	2	7	19	128		0,271	1,351
693	19572	brain	2	1	0	2	3	8	2	3	5	0	4	4	2	3	39		0,375	1,568
690	38933	brain	1	4	1	3	2	18	3	9	5	7	1	1	1	13	69		0,280	1,212
727	35181	brain	2	4	0	0	2	2	9	4	2	3	1	1	1	6	37		0,269	1,386
671	45673	breast	0	9	1	21	1	11	10	7	4	121	0	0	5	10	200		0,447	6,252
673	61040	breast	3	12	1	12	2	24	6	6	2	9	4	2	11	7	101		0,208	1,315
689	28133	brain	5	3	0	1	2	4	6	5	1	2	0	0	0	1	30		0,354	1,481
731	17485	ovary	0	1	2	2	2	2	1	1	1	3	0	2	3	6	26		0,293	1,031
735	42445	ovary	2	5	0	0	4	3	9	4	5	4	3	5	5	2	51		0,313	1,544
737	33675	ovary	3	2	0	0	2	0	3	2	3	0	1	2	4	4	26		0,328	1,275
736	55002	ovary	13	12	2	7	1	3	10	6	5	5	9	4	14	11	102		0,331	1,897
740	65351	prostate	5	14	5	6	9	20	1	7	1	12	1	2	16	14	113		0,373	1,540
686	68626	prostate	8	18	2	1	5	18	14	13	3	28	43	10	9	15	187		0,420	4,321
12 additional normal libraries																				
14799	308589	brain	35	44	5	12	8	44	11	9	15	11	9	33	39	55	330		0,310	3,377
14796	42498	brain	0	6	0	4	0	3	3	1	2	3	0	1	1	11	35		0,249	1,183
14754	33115	breast	5	6	1	1	0	2	3	6	1	0	0	0	4	1	30		0,348	1,505
14756	58181	breast	1	7	1	1	4	7	7	4	5	11	2	1	3	11	65		0,268	1,005
14801	59327	breast	1	7	1	1	4	7	7	4	5	11	2	1	3	11	65		0,268	1,005
14757	79152	breast	2	4	6	6	0	8	6	12	2	2	0	0	9	12	69		0,316	1,653
14798	78288	liver	8	12	9	6	1	17	5	10	7	9	6	3	15	17	125		0,260	1,417
14749	89265	placenta	2	23	6	8	0	27	5	22	6	14	3	4	21	29	170		0,299	1,530
14750	118083	placenta	2	20	6	15	1	26	2	15	9	12	3	4	18	22	155		0,332	1,456
14780	26653	stomach	0	6	1	8	1	7	14	3	1	5	0	0	5	9	60		0,272	1,730
14771	101677	placenta	2	5	4	5	0	12	0	5	5	10	0	1	1	14	64		0,331	1,354
7498	31538	brain	6	2	1	4	1	4	4	0	1	1	3	0	2	4	33		0,321	1,415
68 additional tumor libraries																				
14763	106982	brain	8	15	6	7	6	16	7	9	3	13	2	12	16	22	142		0,231	1,515
14737	105764	brain	4	10	7	14	10	29	2	8	8	22	3	6	13	20	156		0,272	1,458
14765	102439	brain	1	14	1	6	3	14	5	11	6	14	1	6	11	16	109		0,281	1,001
14739	88568	brain	3	4	5	7	4	17	7	10	4	14	5	1	8	23	112		0,212	0,983
14773	118733	brain	10	18	10	16	12	28	0	8	6	17	4	6	12	25	172		0,182	1,320
14766	107344	brain	5	35	2	9	7	29	0	6	14	31	2	2	21	61	224		0,398	2,380
1732	81495	brain	0	18	3	0	1	28	5	6	3	7	1	1	6	20	99		0,303	1,594
14753	49794	breast	0	3	2	2	1	19	6	6	2	3	2	0	4	5	55		0,237	1,476
14748	65314	breast	6	4	2	2	2	7	8	8	2	7	7	1	7	5	68		0,248	1,307
14743	72857	breast	8	1	0	3	1	10	4	8	1	9	3	0	5	5	58		0,301	1,354
14745	81452	breast	1	8	1	1	1	8	1	15	2	8	8	0	7	4	65		0,360	1,825
14747	37435	breast	10	2	0	2	0	6	2	8	0	0	1	0	6	0	37		0,382	2,252
14746	89184	breast	15	7	1	4	0	29	45	42	7	16	12	4	6	9	197		0,409	3,547
14800	50875	breast	3	1	1	4	2	1	1	3	6	1	1	1	1	1	27		0,413	1,606
14797	21951	breast	2	5	0	0	3	3	3	2	4	0	0	0	0	5	27		0,297	1,378
2383	61480	breast	3	11	1	14	2	19	2	7	4	9	1	1	6	13	93		0,246	1,248
1733	70099	breast	1	8	2	10	1	16	4	6	6	13	1	2	6	15	91		0,262	0,968
2382	65045	breast	8	8	2	12	0	6	9	10	13	12	1	3	11	20	115		0,294	1,555
2389	58801	breast	3	1	0	0	0	2	1	3	0	0	0	0	1	1	12		0,289	1,204
1730	61367	breast	3	12	0	7	2	9	7	3	1	6	5	3	3	13	74		0,237	1,108
1731	43902	breast	1	8	4	4	0	15	1	4	0	9	1	1	2	16	66		0,323	1,399
14776	75379	brain	3	16	4	9	4	11	0	10	7	7	4	0	9	13	97		0,293	1,295
1735	74499	brain	1	4	4	4	6	18	0	9	2	5	0	1	7	6	67		0,329	1,590
2408	52659	brain	5	13	2	10	5	15	0	14	5	7	1	3	6	15	101		0,288	1,135
2384	52934	brain	5	7	5	4	5	8	4	14	3	14	0	1	10	16	96		0,252	1,220
14762	68614	brain	3	11	5	11	4	14	0	10	6	10	1	4	7	10	96		0,307	1,220
14740	122690	brain	10	18	4	4	8	28	1	19	5	8	13	0	23	16	157		0,388	2,004
14786	84073	brain	2	17	1	7	10	11	0	11	3	6	0	2	4	12	86		0,320	1,766
14741	120431	brain	14	12	2	6	1	21	2	14	7	7	3	4	15	11	119		0,290	1,603
9103	11582	stomach	1	3	1	3	0	3	6	0	1	1	0	1	0	3	23		0,266	1,300
758	70433	stomach	4	8	2	5	7	14	20	11	6	15	5	0	6	11	114		0,220	1,569
2385	64102	stomach	8	4	1	12	6	13	31	13	8	8	0	0	8	14	126		0,316	2,559
757	66032	stomach	9	10	3	11	6	13	21	9	11	23	5	1	16	22	160		0,229	1,412
9104	15382	stomach	0	5	1	1	6	9	17	1	1	2	2	0	0	1	46		0,450	2,707
14767	100600	brain	3	12	1	8	5	22	2	15	11	12	2	6	13	20	132		0,267	1,209
14768	102322	brain	2	2	2	4	6	24	0	10	0	12	1	0	4	19	86		0,351	1,738
14769	99099	brain	7	13	4	16	9	43	2	10	10	13	9	5	16	25	182		0,253	1,579
1498	62675	brain	9	11	3	8	3	33	2	14	2	17	0	4	8	13	127		0,281	1,527
14807	86887	lung	21	12	4	5	3	18	23	35	9	4	13	0	14	18	179		0,312	2,505
14806	35916	lung	17	4	0	0	3	6	8	14	2	6	4	0	6	4	74		0,351	2,516
14731	52645	brain	1	5	0	11	1	15	5	9	4	7	0	1	7	10	76		0,254	1,186
14788	74295	brain	6	10	3	6	6	11	3	6	1	11	7	3	11	19	103		0,247	1,067
14791	32570	brain	2	5	1	3	1	5	0	4	1	2	1	2	1	5	33		0,217	0,758
14793	89258	brain	4	25	8	14	4	29	5	10	2	20	4	10	8	33	176		0,265	1,603
14774	85984	brain	7	13	3	18	4	28	10	14	4	15	5	7	8	37	173		0,144	1,154
14790	45342	brain	4	10	0	10	3	33	3	16	4	12	2	0	14	16	127		0,244	1,521
14794	74612	brain	3	11	3	1	6	13	6	9	2	21	5	6	0	14	100		0,375	1,522
14781	83671	brain	2	8	1	2	4	10	6	11	5	25	8	16	22	30	150		0,356	2,407
14782	68392	brain	1	4	3	3	4	18	4	20	4	37	10	7	8	21	144		0,346	2,142
14792	59498	brain	2	10	1	9	7	8	3	5	0	3	0	0	2	23	73		0,308	1,796
14783	61853	brain	0	12	0	7	1	8	5	3	4	2	0	1	2	29	74		0,324	2,003
14779	72318	brain	1	16	0	5	2	12	1	4	8	15	1	0	5	22	92		0,337	1,588
14772	60454	brain	5	10	3	4	0	10	0	9	3	6	0	2	3	13	68		0,270	1,155
14787	57469	brain	2	17	2	6	4	20	0	14	8	17	2	4	5	27	128		0,307	1,334
14795	67404	brain	1	9	0	3	3	8	0	17	5	4	0	3	2	6	61		0,315	1,793
14761	85376	brain	1	31	3	16	3	31	1	3	7	5	2	5	3	86	197		0,475	3,693
14734	69971	brain	2	12	3	10	3	19	4	7	2	11	0	2	6	20	101		0,217	1,043
14733	43068	brain	0	14	1	9	1	8	2	5	4	8	1	2	3	7	65		0,252	1,327
14732	48451	brain	5	2	0	2	4	9	1	15	6	12	0	5	4	11	76		0,345	1,587
14775	41338	brain	4	4	0	1	1	1	27	1	15	4	0	2	9	29	98		0,530	3,340
14742	32442	brain	2	2	1	1	0	1	1	4	1	3	1	0	2	7	26		0,231	0,823
743	33957	pancreas	3	2	1	0	1	3	4	1	3	6	3	1	2	0	30		0,329	1,241
744	35750	pancreas	6	2	0	0	2	3	3	5	0	4	2	0	1	1	29		0,330	1,507
7800	34660	stomach	9	8	2	2	3	9	19	4	1	1	9	0	4	3	74		0,435	2,559
8867	43908	stomach	6	10	2	10	10	10	6	2	3	5	4	0	4	3	75		0,322	1,822
8505	32174	stomach	5	10	3	3	6	7	11	1	1	3	5	0	2	1	58		0,453	1,949
14760	51620	stomach	1	10	1	9	0	11	16	7	2	7	4	0	4	13	85		0,281	1,570
1734	51973	universal	3	11	4	24	4	20	15	16	7	18	2	3	7	40	174		0,221	1,633
		total	575	1197	270	817	432	1737	822	1139	542	1413	424	333	964	1859	12524	mean	0,299	1,633
		relative freq.	0,046	0,096	0,022	0,065	0,034	0,139	0,066	0,091	0,043	0,113	0,034	0,027	0,077	0,148	1,000	stand deviation	0,066	0,747

At the start of this study the GEO database (GPL4) consisted out of 154 libraries, of which 60 met our criteria (derived from tissue biopsies and at least 24 tags). At present 254 libraries are available of which an additional 80 meet our criteria. These extra libraries have therefore been used to check whether our previous findings are reproducible. However, since only 12 of the additional 80 libraries were derived from normal tissues a new calculation was performed using all 140 libraries (the 60 original and 80 new libraries), giving totals of 42 derived from normal tissues and 98 derived from tumor tissues. The observed tag counts are shown in Table 3. Expected frequencies for the tag counts of the 14 genes of a library were obtained with the overall expression profile derived from the sums of the tag counts in all 140 libraries (Table 2 C). The expected and observed tag counts were again compared by chi-square. A comparison of the chi-squares of the normal and tumor tissues by Wilcoxon's test revealed a much greater significant difference between the two groups then previously observed (P = 1.65 × 10^-8 ^instead of P = 1.73 × 10^-6^). This shows that the 80 new libraries display a similar difference between normal and tumor tissues to that observed with the 60 libraries used previously.

Of these 140 libraries, 30 were derived from breast tissue, 11 of these originating from normal breast. These normal and tumor breast tissues were compared separately to deal with the potential disadvantage posed by tissue heterogeneity. Expected frequencies for the tag counts were again obtained with the overall expression profile derived from the sums of the tag counts in these 30 libraries (Table 2 D). The expected and observed tag counts were again compared by chi-square. Comparison of the chi-squares of the normal and tumor breast tissues by Wilcoxon's test (P = 0,0033) revealed a similar difference between normal and tumor to that found for all tissues.

### Variation in transcription specific or unspecific?

Comparison of the expression profile of the 37 most orderly libraries with that of the 37 most disorderly libraries shows that these profiles are rather similar (Table 2E, F). This indicates that excessive variation in transcription is multi-directional and does not lead to a specific and systematic change in the expression profiles of all tissues. However for individual tissues this still could be the case.

### Heritability of variant profiles

**Figure 1:**
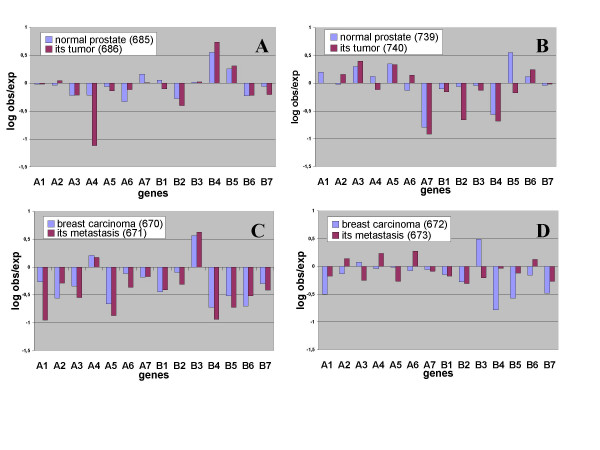
Comparison of deviation profiles of clonal derivatives from normal (A and B) or tumor tissues (C and D). Significance of the correlations: for A: R = 0,77 and P = .0,00135, for B: R = 0,73 and P = 0,00329, for C: R = 0,84 and P = 0,00016 and for D: R = 0,04 and P = 0,887

That the observed variation in transcription is not just due to momentary fluctuations in transcription rates follows from an analysis of pairs of libraries present in the database; i.e. pairs from both tumor and normal tissue or from both tumor and metastatic tissue, each pair being derived from the same individual. Of the six available library pairs, four have enough tags for a detailed analysis of deviation in the expression of each individual proteasome gene. By taking the log of the ratio of "observed tags"/"expected tags" for each gene, a profile is obtained that shows the degree of aberration in expression for each proteasome gene. The data presented in Figure 1A reveal a high correlation between the abnormal expression pattern of a normal prostate and its tumor (R = 0,77 and P = 0,00135). This indicates that a deviating expression profile can be extremely stable and can be transmitted to a clonal derivative as a constitutive trait.

Essentially the same picture is seen in Figure 1B, where a high correlation exists between the deviant profile of another prostate tumor and the normal prostate tissue from which the tumor was derived (R = 0,73 and P = 0,00329). The expression profiles of the two prostates clearly differ from each other, which suggests that a specific expression profile for the prostate does not exist and that these two profiles are deviating dissimilar from the mean expression profile.

The similarity in deviating expression profiles of a normal tissue and its tumor suggests the possibility of a disorderly condition in the normal tissue being a predisposing factor in the eventual appearance of the tumor.

The same persistence of a deviating expression profile is observed for the correlation of a breast tumor and its metastasis as shown in Figure 1C (R = 0,84 and P = 0,00016).

Although heritability of deviating profiles is indicated for these three instances, a direct proof for disorder as cause for these deviations is absent.

### Both progression and regression in degree of variation occurs

Inspection of the three profiles of the clonal derivatives that correlated significantly with the three profiles of the tissues of origin (Figure 1A, B and 1C) indicates that 11 genes progressed to further deviation and 3 to less variation (only those genes were counted that had a log obs/exp that is larger than 0.5 or -0.5 which represents a 3.2-fold over-expression or under-expression respectively). As this difference is significant (P = 0,032) it suggests that the deviation in expression is progressing in clonal derivatives. The figure further indicates that progression of a deviating expression profile generally occurs in small steps, but that larger jumps may also occur (PSMA4 in Figure 1A).

That progression does not always occur is suggested by Figure 1D in which no significant correlation is observed between a tumor and its metastasis (R = 0,04 and P = 0,887) and in which a decrease in deviation is apparent in the metastasis. This figure therefore indicates that the deviating profile does not always persist in a clonal derivative. The metastasis actually exhibits an expression profile that is almost indistinguishable from normal, thereby suggesting that the changes in expression were non-genetic in origin.

Apparently, spontaneous epigenetic modifications that interfere with normal gene expression patterns can occur. At present, the cause of these modifications remains obscure. Changes in transcription factors, DNA methylation patterns [14], unusual DNA structures [15], alterations in nuclear organization [16], interference by noncoding RNAs [17] or changes in the macromolecular transcriptional apparatus [18] might be involved. Possibly all factors known to influence transcription rates could be involved. Among these factors alteration in nuclear organization is very attractive as it easily explains the simultaneous changes in expression in a number of genes and as numerous nuclear constituents could be involved. If so these findings would be of importance to the rapidly developing field of "spatial nuclear organization as a structural component in gene expression" [19,20].

### Under-expression and over-expression in deviating libraries

In the first set of 60 libraries, 15 variant libraries were identified that deviated strongly (P < 0,01) from the overall profile. The changes in the degree of transcription of the individual genes in these libraries were expressed as the log of the ratio of observed and expected tags (Table 4 A). In the second set of 80 libraries, 22 variant libraries were identified (Table 4 B).

**Table 4:** Deviation in transcription (log obs/exp) in the individual genes of the 37 most disorderly libraries.

A. The 15 most disorderly libraries identified in the first set of 60 libraries.																
		log obs/exp														
	gene	PSMA1	PSMA2	PSMA3	PSMA4	PSMA5	PSMA6	PSMA7	PSMB1	PSMB2	PSMB3	PSMB4	PSMB5	PSMB6	PSMB7	deviation index
GSM																
671		-0,954	-0,288	-0,552	0,172	-0,872	-0,364	-0,169	-0,412	-0,309	0,625	-0,938	-0,727	-0,513	-0,414	0,431
686		-0,015	0,048	-0,216	-1,115	-0,137	-0,115	0,013	-0,107	-0,398	0,025	0,731	0,309	-0,222	-0,202	0,399
672		-0,497	-0,133	0,080	-0,041	-0,018	-0,075	-0,059	-0,147	-0,279	0,489	-0,783	-0,571	-0,154	-0,481	0,317
687		-0,171	0,193	-0,071	-0,669	-0,390	0,474	-0,688	0,401	-0,430	-0,977	-0,457	-0,245	-0,254	-0,234	0,409
699		-0,485	-0,297	-0,084	-0,682	0,074	-0,092	0,414	-0,487	0,160	-0,989	0,609	0,043	0,257	0,009	0,438
745		-0,299	-0,111	-0,199	0,048	-0,519	0,410	-0,038	-0,426	-0,558	-0,406	-0,585	-0,373	0,220	0,115	0,311
786		0,032	-0,132	-0,044	-0,040	-0,364	0,005	-0,661	-0,271	-0,102	-0,348	-0,430	0,480	-0,102	0,474	0,315
574		-0,171	0,052	-0,071	0,309	-0,691	0,005	-0,143	-0,377	-0,731	-0,074	-0,059	-0,245	-0,129	0,323	0,305
760		0,323	-0,104	-0,317	-0,012	-0,335	0,261	0,067	0,301	-0,198	-0,745	0,075	-0,491	0,024	-0,275	0,312
736		0,459	0,135	0,048	-0,006	-0,573	-0,630	0,130	-0,180	0,087	-0,460	0,315	0,174	0,233	-0,074	0,329
1497		0,275	0,111	-0,102	-0,399	0,277	-0,178	-0,719	-0,204	0,493	-0,054	-0,488	0,422	0,016	-0,010	0,347
762		-0,836	0,051	0,343	0,289	0,090	0,143	-0,751	-0,140	-0,016	0,001	-0,821	-0,007	-0,095	0,065	0,392
765		0,053	-0,361	-0,148	-0,445	-0,166	-0,302	0,349	0,102	0,396	-0,054	-0,534	-0,322	0,234	-0,010	0,291
740		0,000	0,158	0,401	-0,118	0,337	0,150	-0,915	-0,157	-0,657	-0,125	-0,684	-0,171	0,246	-0,013	0,395
739		0,198	-0,025	0,298	0,126	0,346	-0,130	-0,796	-0,106	-0,061	-0,044	-0,565	0,549	0,115	-0,041	0,347
mean		-0,139	-0,047	-0,042	-0,172	-0,196	-0,029	-0,264	-0,147	-0,173	-0,209	-0,308	-0,078	-0,008	-0,051	
standard deviation		0,413	0,175	0,252	0,406	0,380	0,291	0,446	0,254	0,361	0,462	0,522	0,400	0,229	0,252	
B. The 22 most disorderly libraries identified in the second set of 80 libraries.																
	gene	PSMA1	PSMA2	PSMA3	PSMA4	PSMA5	PSMA6	PSMA7	PSMB1	PSMB2	PSMB3	PSMB4	PSMB5	PSMB6	PSMB7	
GSM																
14761		-0,956	0,218	-0,153	0,086	-0,353	0,056	-1,116	-0,775	-0,084	-0,646	-0,525	-0,028	-0,702	0,470	0,475
14746		0,218	-0,430	-0,632	-0,519	-0,833	0,025	0,535	0,369	-0,086	-0,143	0,251	-0,127	-0,403	-0,512	0,409
14799		0,364	0,146	-0,155	-0,263	-0,152	-0,016	-0,299	-0,521	0,023	-0,528	-0,095	0,568	0,188	0,052	0,310
14775		-0,059	-0,377	-0,335	-0,824	-0,536	-1,140	0,610	-0,957	0,541	-0,449	-0,531	-0,131	0,069	0,292	0,530
7800		0,418	0,049	0,091	-0,398	0,066	-0,061	0,582	-0,230	-0,510	-0,926	0,548	-0,307	-0,158	-0,568	0,435
14806		0,683	-0,263	-0,222	-0,710	0,054	-0,249	0,195	0,302	-0,220	-0,159	0,184	-0,319	0,007	-0,454	0,351
14807		0,396	-0,164	0,002	-0,390	-0,324	-0,150	0,275	0,322	0,055	-0,714	0,318	0,002	-0,003	-0,179	0,312
2385		0,131	-0,487	-0,446	0,145	0,131	-0,137	0,559	0,046	0,158	-0,258	-0,642	-0,242	-0,092	-0,134	0,316
14781		-0,536	-0,252	-0,511	-0,699	-0,110	-0,317	-0,220	-0,092	-0,112	0,171	0,196	0,596	0,282	0,131	0,356
14766		-0,314	0,213	-0,386	-0,222	-0,043	-0,030	-1,174	-0,531	0,159	0,088	-0,582	-0,483	0,085	0,263	0,398
14782		-0,819	-0,535	-0,016	-0,505	-0,092	-0,044	-0,378	0,185	-0,191	0,359	0,311	0,255	-0,140	-0,006	0,346
14740		0,160	0,098	0,088	-0,401	0,188	0,128	-1,001	0,143	-0,115	-1,230	0,404	-0,611	0,298	-0,145	0,493
14783		-0,553	0,208	0,074	0,129	-0,428	-0,129	-0,015	-0,372	0,075	-0,642	-0,423	-0,324	-0,476	0,400	0,324
8867		0,236	0,140	0,085	0,295	0,583	-0,021	0,076	-0,537	-0,038	-0,233	0,190	-0,313	-0,163	-0,574	0,322
14745		-0,481	0,105	-0,155	-0,643	-0,356	-0,057	-0,641	0,399	-0,153	0,033	0,552	-0,252	0,141	-0,387	0,360
14792		-0,241	0,140	-0,216	0,250	0,428	-0,118	-0,226	-0,139	-0,515	-0,455	-0,412	-0,313	-0,465	0,311	0,308
14795		-0,467	0,169	-0,141	-0,153	0,135	-0,044	-0,628	0,467	0,258	-0,255	-0,337	0,239	-0,390	-0,198	0,315
14786		-0,305	0,307	-0,280	0,077	0,519	-0,044	-0,766	0,140	-0,102	-0,217	-0,476	-0,076	-0,227	-0,035	0,320
14768		-0,309	-0,627	0,016	-0,171	0,293	0,290	-0,771	0,093	-0,584	0,079	-0,480	-0,382	-0,232	0,159	0,351
14780		-0,460	0,000	-0,135	0,280	-0,335	-0,095	0,525	-0,279	-0,434	-0,151	-0,330	-0,231	0,015	-0,015	0,272
14757		-0,217	-0,234	0,586	0,097	-0,393	-0,095	0,099	0,265	-0,191	-0,607	-0,388	-0,289	0,212	0,052	0,316
1734		-0,425	-0,178	0,026	0,316	-0,175	-0,080	0,114	0,006	-0,030	-0,036	-0,471	-0,196	-0,280	0,192	0,221
mean		-0,161	-0,080	-0,128	-0,192	-0,079	-0,106	-0,167	-0,077	-0,095	-0,314	-0,124	-0,135	-0,111	-0,040	
standard deviation		0,432	0,284	0,260	0,367	0,358	0,261	0,580	0,402	0,265	0,382	0,409	0,308	0,269	0,314	

If one considers the data In Table 4 as a whole, 331 under-expressions are observed against 187 over-expressions. Thus under-expression predominates (P = 2,5 × 10^-10^) in deviating libraries. There is no evidence that differences exist between the genes in their frequencies of under- and over-expression, since the chi-square for heterogeneity is not significant (P = 0,539). However differences between the genes in the degree of under- and over-expressions do exist. The 14 standard deviations of the log obs/exp values, calculated for each individual gene from the 15 most abnormal libraries as shown in Table 4 A, were found to be heterogeneous when compared by the test of Bartlett (P = 0,002). The standard deviations of the 22 most abnormal libraries in Table 4 B correlate with those of Table 4 A (P = 0,022) thus showing a similar pattern of differences between genes. For the whole set of 37 deviating libraries the 3 most variable genes are PSMA7, PSMB4 and PSMA1, while the 3 least variable genes are PSMA2, PSMB6 and PSMA3.

### Epigenetic origin of excessive variation in transcription

At first sight it does not seem surprising that cancer tissues show greater variation in transcript abundances, as tumor cells are usually aneuploid. However excessive variation in expression profiles is observed in normal tissues as well. This holds not only for the two normal prostates mentioned previously but also for other normal tissues e.g. cortex (GSM 786), normal retina (574), normal breast (760), normal colon (728), normal brain (676) and normal lung (762). Moreover tumors with a normal expression profile are not rare although tumors, as a rule, are known to be aneuploid and carry mutational damage in oncogenes. In addition the similarity in deviating expression profiles of pairs of normal and tumor prostate tissue (supposedly the first being diploid and the second aneuploid) also indicates a non-genetic origin of the deviating profiles Loss of deviation in a metastasis is, similarly, also suggestive of a non-genetic origin. Therefore it would appear that aneuploidy, as such, or mutated oncogenes do not play predominant roles in the emergence of excessive variation in transcription and thus that there is an epigenetic origin.

### An index for the degree of deviation in transcription

The degree of deviation can be quantified in a deviation index by taking the standard deviation of the log obs/exp values from the 14 genes in a library (log ratio deviation index) or the standard deviation of the z-scores (z-score deviation index). The log ratio deviation index will be suitable to reflect fold-changes in expression, while the z-score deviation index wiil be more suitable to reflect percentual changes in expression.

If the log ratio deviation index and the z-score deviation index were calculated for all 140 libraries (shown in table 3) and then normal and tumor libraries were compared with the test of Wilcoxon, tumor libraries were again found to be more deviating than normal libraries. For the log ratio deviation index the significance is considerable (P = 3.6 × 10^-6^) while for the z-score deviation index the significance is much less (P = 0,0177) indicating that the changes in expression reflect fold-changes rather than percentual changes.

This deviation index provides a means by which to test whether differences in degree of deviation exist between tumors derived from different tissues. To this end a table was prepared with the log ratio deviation index of 5 tumors (astrocytoma, breast cancer, ependymoma, gastric cancer and medulloblastoma), each tumor represented by 9 libraries (Table 5). When compared with ANOVA (Analysis Of VAriance), the degree of deviation was not influenced by tumor type (P = 0,387). Therefore tumors do not appear to differ systematically in degree of deviation.

**Table 5:** No heterogeneity in deviation index in 5 types of tumors, 9 libraries per type of tumor (P = 0,387).

	replication								
tumor type	1	2	3	4	5	6	7	8	9
Astrocytoma	0,231	0,229	0,313	0,272	0,281	0,212	0,182	0,398	0,303
breast carcinoma	0,248	0,409	0,246	0,262	0,294	0,327	0,375	0,237	0,323
Ependymoma	0,293	0,331	0,288	0,252	0,307	0,388	0,320	0,290	0,281
gastric cancer	0,266	0,220	0,316	0,229	0,450	0,435	0,322	0,453	0,281
Medulloblastoma	0,254	0,247	0,265	0,144	0,244	0,375	0,356	0,346	0,308

### Deviation of individual genes is often not independent

**Figure 2:**
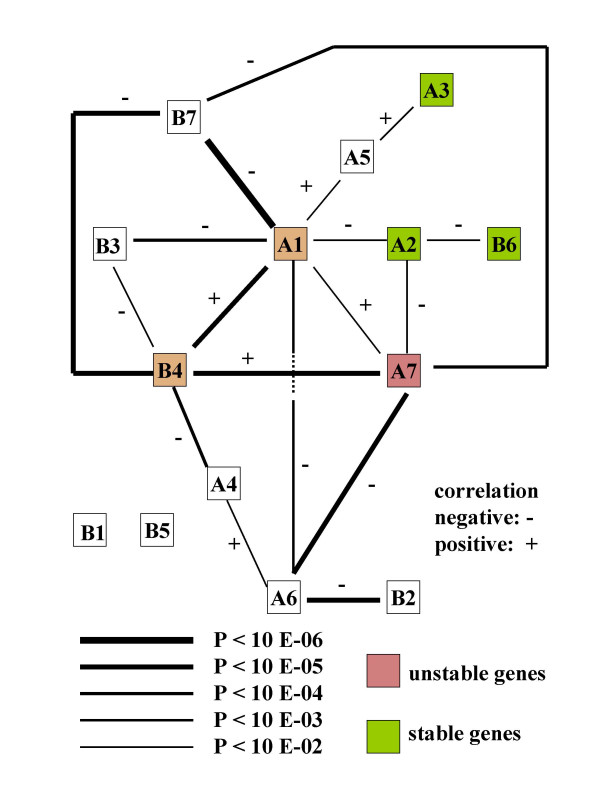
Network of significant correlations between the deviations in expression (log obs/exp) of the 14 proteasomal genes.

Correlations between the values of log obs/exp in the 140 libraries were calculated for each pair of genes to determine whether the deviation of the individual genes is independent from each other. Of the 91 correlations, 18 significant correlations (P < 0,01) were observed. These significant correlations are shown In Figure 2. This figure shows a rather simple network of links in deviation of individual genes. Both positive and negative correlations were seen. The existence of these interactions suggests that there will be patterns in the emergence of deviant profiles.

The 3 most unstable genes are involved in significant correlations on 17 occasions as compared to 5 occasions for the 3 most stable genes. This indicates that instability in expression and involvement in correlation are somewhat related (P = 0,0105). The degree of expression of the individual genes (see Table 2) does not seem to be involved in this pattern of interactions. Changes in the spatial architecture of the nucleus could well be responsible for the observed dependence, as any change in architecture will affect many genes.

### Possible significance of the observed variation in transcription

As a whole, tumor tissues demonstrate a much larger variation in transcription profiles than normal tissues. This suggests, therefore, that the observed excessive variation in expression of proteasome genes is due to disorder and is not a consequence of an orderly regulation. However one cannot exclude that alternate hypotheses are possible. If due to disorder it is at present still to early to characterize the sources of the disorder observed. Although mutational damage could, in theory, be involved, this is not supported by the data as discussed above. Consequently, noise and/or chaotic processes might be causally involved but it is still too early to make any decisive statement in relation to this.

The observed deviation appears to be due to stable structural epigenetic changes. If our assumption is correct; that all tissues initially have an approximately similar expression profile (which is supported by the grosso modo similar expression profile of orderly and disorderly libraries, see Table 2 E and F), then the findings suggest that this expression profile can be altered in a progressive and unpredictable way resulting in widely different expression profiles. In addition, degree of progression of this deviation seems to have some unpredictability, as it was observed to exert a small effect on a number of genes simultaneously or a major effect for just one gene in particular (Figure 1). Therefore a deterministic epigenetic process could well be the cause for the observed deviations. Only time will tell.

Although the deviations in transcription, as described in this paper, relate only to the transcription of the proteasomal genes, it is not illogical to suppose that similar deviations will exist in transcription profiles of genes involved in other functions of the cell and that the corresponding deviation indices could provide information on the degree of order in transcription.

Our present working hypothesis for the structural aspect of the observed variation is that the patterns of over- and under-expression are a reflection of the localization of the genes within the nucleus. If this hypothesis were correct, then one would expect that future research would reveal correlations between genes that are completely unrelated in function in terms of their degree of over- or under-expression. Consequently, the next desirable step will be to investigate whether similar deviating profiles can be found in other organelles and/or pathways and whether a deviation in one profile corresponds with deviation in another profile.

This phenomenon of deviation in transcription could provide a new method to study genetic dysfunction. Apart from the field of gene expression, research into this phenomenon could turn out to be of value in other fields:

#### 1. Genomic instability

Any decrease in the ability of a cell to carry out normal cellular functions could lead to a less efficient DNA replication and to increased production of free radicals, resulting in a greater degree of spontaneous DNA damage. As DNA repair could also be less efficient a higher spontaneous and induced mutation rate might result. Therefore this new phenomenon of variation might well underlie the hitherto unexplained phenomenon of "persistent delayed genomic instability " [2] and might also provide an explanation for the trans-generational effects of parental irradiation [21].

#### 2. Carcinogenesis

Mutations in oncogenes predispose a cell to developing a transformed phenotype. The switching on of the telomerase gene and other genes involved in immortalization is another prerequisite step in the process of carcinogenesis. In fact the acquisition of an immortal phenotype is the rate-limiting step in carcinogenesis [6,22]. The course of these epigenetic events, ultimately resulting in malignant transformation, is still not understood. Progressive disorder in transcription in pre-cancerous lesions could be involved in the rare switching on of genes involved in immortalization.

#### 3. Cellular aging

The current database does not contain enough information to determine whether disorder plays any role in cellular aging. However as progressive epigenetic changes could well be at the core of cellular aging it is not inconceivable that the aging process will be reflected in the degree of deviation in expression profiles. Whether this will be seen as an increase or a decrease in variation is a fascinating question. As cellular aging is still largely a black box, investigation of the role of disorder during aging might contribute to an improved understanding of this process.

#### 4. Cell dynamics

The cell is a complex system. It is a mystery as to how all of the cellular subsystems of the cells interact and function collectively as a complex whole. In complex systems 'spontaneous" processes like pattern formation, oscillation, bifurcation and chaotization occur, These processes might depend on very simple rules[23]. The observed deviations in transcription might reflect chaotic processes. So far however there is no direct evidence that the observed deviations have anything to do with chaos, only time will tell. Nevertheless, whether chaotic in nature or not, the study of variation in the regulation of gene expression might contribute to a better insight into the cell as a complex system, especially if it reflects changes in the spatial organization of the nucleus.

#### 5. Practical implications

One direct consequence arising from this study is that determination of the degree of deviation can serve as a control for the quality of libraries, which are to be used for the identification of genes involved in cellular processes. Deviating libraries will be less suitable for the identification of the genes involved.

A deviation index might further prove to be of prognostic value in predicting the probability of progression of neoplastic and possibly of pre-neoplastic lesions and likewise could be used as an indicator of health.

In addition, since deviation in gene expression can either increase or decrease, it would be useful to determine the effects of medication, promoters and anti-promoters on the degree of deviation.

In many respects, the data presented in this paper should be considered as very provisional. Ideally instead of only the 14 genes studied here, one would like to see comparative data for a few hundred genes as well as the use of greater numbers of large libraries from both healthy and unhealthy donors. Although such information is not yet available, this could soon be the case. Nevertheless the data obtained so far does indicate that the study of variation in transcription in the cell could provide new clues in biology and biomedicine.

## Methods

### Gene expression

The tags used to determine the degree of expression of the proteasome genes in SAGE libraries were derived from the GEO database [24] the proteasome tags in SAGE libraries were found by importing both the tags of table 1 as well as the library tag count file into Microsoft Access. A query that joins both tag fields results in a table showing the abundance of tags for each proteasome gene.

The SAGE libraries were obtained from the "Gene Expression Omnibus" (GEO) [25].

The 30 libraries from normal tissues are: GSM number 572, 573, 574, 676, 677, 685, 688, 691, 692, 695, 708, 713, 719, 728, 729, 738, 739, 760, 761, 762, 763, 780, 781, 785, 786, 819, 824, 1499, 2386, 3242. The 30 libraries from cancer tissues are: 670, 671, 672, 673, 686, 687, 689, 690, 693, 696, 697, 698, 699, 727, 731, 735, 736, 737, 740, 745, 755, 756, 765, 792, 793, 1497, 1516, 2443, 2451, 2578 (Table 3). Only one library (GSM 709, leukocytes) was excluded since libraries derived from blood and blood-forming tissues might express the immunoproteasome that might then interfere with expression of the 20S proteasome. The 80 additional libraries are: 743; 744; 757; 758; 1498; 1730; 1731; 1732; 1733; 1734; 1735; 2382; 2383; 2384; 2385; 2389; 2408; 7498; 7800; 8505; 8867; 9103; 9104; 14731; 14732; 14733; 14734; 14737; 14739; 14740; 14741; 14742; 14743; 14745; 14746; 14747; 14748; 14749; 14750; 14753; 14754; 14756; 14757; 14760; 14761; 14762; 14763; 14765; 14766; 14767; 14768; 14769; 14771; 14772; 14773; 14774; 14775; 14776; 14779; 14780; 14781; 14782; 14783, 14786, 14787, 14788, 14790, 14791, 14792, 14793, 14794, 14795, 14796, 14797, 14798, 14799, 14800, 14801, 14806 and 14807

### Statistics

The observed frequencies of the expression profiles were compared with their expected frequencies by chi-square. As the lower limit of the proteasomal tag count per library was 24 and the number of genes was 14, the expected number of tags was often less than 5 which is the lower limit of reliability for the application of chi-square. Therefore the outcome of the chi-square test was only used in a parameter free test (Wilcoxon) to compare the group of normal tissues with the group of cancer tissues.

For the calculation of the deviation index the standard deviation of the log of observed tags/expected tags was used. In those cases where the observed number of tags was 0, it was assumed that there was 1 tag. This index has as disadvantage that it is only symmetric if the changes in expression occur as fold-change. If the variation in expression occurs in percentages this index is not symmetric. Therefore a second index was calculated based on z-scores (z-score = (obs - exp)/√exp). Both deviation indices, noted as log ratio deviation index and z-score deviation index, have been applied.

The deviation index of 5 types of tumors was compared with ANOVA. In order to have 9 tumors per group some libraries had to be omitted from the analysis. This was achieved by leaving out the libraries with the lowest number of counts.

## Reviewers' comments

### Reviewer's report 1

Trey Ideker, University of California San Diego, La Jolla, California, United States

This manuscript by JWIM Simons examines the mRNA levels of proteasomal proteins across publicly-available SAGE data from both cancer and normal tissues. It reports that proteasomal mRNAs show more variance away from their average levels when looking in cancer tissues versus in normal tissues. It also presents a corollary finding, that normal and cancer tissues taken from the same patient tend to have protesomal expression levels that are very similar. Finally, the manuscript makes speculative remarks about the possible interpretation and impact of these findings. In this regard, the main claim is that high variance in proteasomal RNA levels is indicative that the cell is in a "disorderly state" and that this disorderly state is likely a cause, not an effect, of cancer.

The basic finding, that proteasome mRNA levels as measured by SAGE have higher variance from the mean when looking in cancer cells, is interesting and, as far as this reviewer can tell, arrived at through reasonable use of statistical methods. The corollary finding, that mRNA levels from the same patient are correlated in cancer versus normal cells, is also interesting and is nicely controlled by comparing the correlation within versus between patients.

On the other hand, framing these findings within an argument that cellular transcription can be "ordered" or "disordered" is speculative at best and much less compelling. In order to support the "disorder" argument (which is not a small suggestion in the discussion, but also appears in the manuscript title, abstract, introduction, and results) a larger body of evidence would need to be examined and presented. For instance, perhaps a more likely null hypothesis is simply that cancer cells are proliferating and thus have more protein turnover. And there are other equally plausible ideas that do not relate to a global order vs. disorder phenomenon. Without examining such alternate hypotheses and addressing them, the article reads much more like a "commmentary" or "opinion" article than a primary research paper.

#### Author response

*Surely so far there is only an indication and not yet a proof that cellular transcription can be disordered. At present there is also not yet an overview of all possible alternate hypotheses let alone to examine them. To make sure that disorderly transcription is only one possible explanation a sentence has been added that states that alternative explanations are possible*.

There is also a semantic problem with the use of the term "disorder". Ordered versus disorder has a concrete (and very different) meaning in the field of information theory, which attempts to measure it through quantities such as entropy. In fact, entropy might have been a much more natural metric to use for the proposed disorder index.

#### Author response

*In order to have a better separation between findings and speculation the term disorder has been replaced by a more descriptive term (e.g. excessive deviation) in the case of findings. This, I hope, dissolves the "semantic problem" and improves the distinction between fact and speculative interpretation. The changes have been made throughout the text. Your remark that "entropy might have been a much more natural metric to use for the proposed disorder index" has raised my interest greatly but I have no idea how such a thing could be accomplished*.

A key assumption of the paper is that for organelles such as the proteasome, "the products of the genes involved [are] available in the correct amounts" and "therefore we can assume an optimal expression pattern of the transcription of the genes in question exists" (Results and discussion section, first paragraph). This may not need be the case. For instance, for the ribosome it has been shown that, while RNA levels can fluctuate in response to stimuli, the overall ribosomal protein levels are buffered from change. Depending on the function/component, such buffering can be due to differential RNA degradation, protein translation, and so on.

#### Author response

*Probably there will be many mechanisms within the cell that can buffer undesirable fluctuations. This does not bring down the assumption that optimal functioning will depend on optimal conditions and to some extent this also should hold for an expression profile*.

Moreover, given the assumption that correct amounts of each proteasomal subunit are needed, then why are the average or "expected" amounts of each subunit so different from one another in the SAGE data? Since the main result is measuring deviations from these expectations in individual patients, this point is particularly important. I would be interested to see how the results are impacted if the expected amounts are equal to each other across all subunits.

#### Author response

*You rightly put the finger on the remarkable differences in tag counts between genes in the mean expression profile while all subunits are equally important for building the proteasome and thus similar frequencies are expected. For this there is not yet a definite answer: remarkable is that when another tag is used (for some genes an additional tag is available) the absolute frequency can be consistently much lower. Explanations could be the length of the cDNA or the efficiency of the cutting enzyme, maybe also splicing or RNA degradation?? For this point I have been looking to microarray data and found that the mean profile is very different from the SAGE profile, the two profiles did not even correlate. Apparently the technique to obtain expression data affects the outcome and it is not clear to me whether this heterogeneity is biological. Taking the mean of the two expression profiles of microarray and SAGE produces an expression profile that is much closer to the "equal amounts" concept. Also this is an issue for the future*.

In conclusion, my recommendation for this article would be to (1) remove many of the speculative remarks, or at least leave them for the discussion, including any interpretation of the results as "disorderly"; and to also (2) perform and present a more comprehensive body of findings which support the points in the discussion section that remain. For this second point, at minimum it would be nice to see a survey of all organelles/functions in the cell and whether they show greater variance in cancer than normal. Otherwise there is no evidence that the specific anecdote of the proteasome can be abstracted to some general principle of the cell.

#### Author response

*these recommendations have been met by the clearer distinction between findings and possible interpretations as described above*. We *surely agree that the findings with the proteasome cannot yet be abstracted to a general principle. Therefore *w*ith respect to your recommendation to make a similar analysis for all organelles/functions in the cell, it is obvious that such would be our wish and this was as such also stated in the manuscript. However this is physically impossible, my lifespan would not be long enough. The only thing I can do is to point to this new phenomenon and to contribute further to its interpretation in the hope that also other scientists will study this new phenomenon*.

### Reviewer's report 2

Itai Yanai, Harvard University, Cambridge, Massachusetts, United States

In this paper, Simons uses public SAGE data to quantify changes in gene expression of the set of 14 genes that compose the proteasome. First, the overall relative frequencies of these genes are calculated. A SAGE library is then described as disorderly if the standard deviation of the genes' observed to overall differences is high. It is noteworthy, that the frequencies of the most disorderly libraries are remarkably similar to those of the most orderly libraries, suggesting there is no characteristic state of disorder but instead that each mess is unique.

Simons then shows that the overall tumor libraries are significantly more disorderly – as evidenced by Wilcoxon's test – as a group than the normal libraries. Since this is an important point, I believe it would be helpful to visualize this difference with a principal components type analysis. The 14 dimensions (genes) can be reduced to 2 or 3 and plotted for both the normal and tumor samples. This method is further called for since the author shows in Figure 2 that the genes expression are correlated.

#### Author response

*No doubt this could be worthwhile. However, the analysis is unfamiliar to me (I had even never heard of the method). In a first trial with PCA it was found that the first three factors count for only 41,46% of the variability and thus principal components do not appear to be present. In the future, when I am more familiar with this method, I certainly will try to perform a PCA*.

An issue is next raised about whether disorderly profiles represent "momentary fluctuations in transcription rates" or heritable states. The author shows that deviations of the proteasome genes' expression in a tumor correlates with those in non-tumor from the same patient. Based upon this evidence, the author states that "a disorderly expression profile can be extremely stable and can be transmitted to a clonal derivative as a constitutive trait." However, since this result was observed in three of only four instances, it would be prudent not to draw too strong of a conclusion about the stability of gene disorder based upon this dataset alone. Furthermore, one could argue that difficulties associated with exclusively dissecting tumor vs. non-tumor samples from a given tissue, compromise our ability to meaningfully compare them; i.e. the two may be similar simply because of impure sample isolations.

#### Author response

*Hereditability of deviating expression profiles was observed for three of the four cases. To me it seems unlikely to be due to admixtures of normal tissue in three tumor samples. Of course so far there are only three cases. To stress that this hereditability is not necessarily connected with disorder in transcription a sentence has been added and a question remark has been added*.

The author presents an index for disorderliness: standard deviation of the log obs/exp values. I would advise against this formulation because it is not symmetric, biasing in favor of reduced expression. For example, an increase of expression by 10% would be log(1.1/1) = 0.0952, while a decrease by 10% results in log(0.9/1) = -0.1054. Since, the author makes the point that disorderliness tends to occur in terms of under-expression, the lack of symmetry in the index is a clear confounding effect. This can be easily fixed by taking the log of the absolute difference, and adding a negative sign if obs is less than exp. However, it may be the best to convert to Z-scores, to explicitly take into account the variation of each gene's expression.

#### Author response

*Your remark on the possible absence of symmetry in the log ratio deviation index did initially worry me. According to your suggestion z-score values have been determined and compared to the log ratio. It turns out that the z-scores discriminate less between normal and tumor indicating that the changes in expression reflect fold-changes rather than percentual changes. This has been added to the text*.

### Reviewer's report 3

Stephan Beck, The Wellcome Trust Sanger Institute, Hinxton, United Kingdom

I accepted to review this manuscript on the premise that experts in SAGE expression analysis and statistics will be secured as additional reviewers to assess the methodologies and tests carried out in this study.

The manuscript by JWIM Simons aims to address several fundamental questions, listed on page 2:

1) Can disorder in transcription be demonstrated?

2) Does such disorder have a degree of permanence?

3) Does it play any role in health and disease?

While I commend the author for tackling such complex questions, I do not agree with many of the conclusions and believe the study is compromised by inadequate assumptions and data selection. My main concerns are:

1) The selection of only 14 genes (20S proteasome complex) is not nearly enough to represent the human transcriptome. In addition, I do not agree with the rationale for some of the additional stratifications of the libraries/data given in the 'Results and Discussion' (first section), and in the 'Methods'. For example, leukocyte libraries were excluded from the analysis on the basis that "these cells might express the immunoproteasome PSMB8 and PSMB9 genes that might then interfere with expression of the 20S proteasome". According to the GNF gene expression database , PSMB9 for instance is not only expressed in blood, but also in lung, thymus, spleen and heart. Therefore, why were these tissues not excluded as well if this is the right thing to do in the first place?

#### Author response

*I agree completely with your remark that a sample of 14 genes is not nearly enough to represent the human transcriptome. This has also been stated in the paper. This sample is just the first start to investigate whether such an approach is possible and could be useful. With respect to selection criteria, a selection has to be made, as the immunoproteasome is another organelle than the proteasome. The point therefore is where to draw a line. Whether the immunoproteasome could be normally present in some tissues that are not involved in blood-formation I really do not know. As these expressions, if present, are very low the best policy, in my view, is to draw a line between blood-forming tissues and others. Therefore for the selection of the libraries all libraries from blood forming tissues would have excluded, thus also spleen, thymus and tonsils*.

2) I could not work out how the author defines disorderly expression profiles, except for the definition on page 2 where 'disorder' is defined as "excessive variation in transcription irrespective of cause". However, the author does not seem to take into account that natural variation in gene expression can be quite high (up to 14.13%) in unrelated individuals as compared to e.g. monozygotic twins (up to 1.76%) (see e.g. Sharma et al. Physiol. Genomics 2005 21:117-23). If the above ~10-fold difference falls within the definition used here for 'excessive', then perfectly 'normal' expression profiles would be classified as 'disorderly'.

#### Author response

*I also agree that at present it is not possible to conclude with certainty what to classify as disorderly. Therefore in order to have a better separation between findings and speculation I have been replacing the term disorder by more descriptive terms (e.g. excessive deviation) in the case it concerns findings. The changes have been made throughout the text and a question remark has been put in the title. Your argument about natural variation in unrelated individuals is strictly speaking not fully valid as in that case the quantitative expression of genes was compared and no use was made of an expression profile, which shows the relative expression*.

3) I do not agree with the 'one-fits-all' assumption made on in the 'Results and Discussion' (first paragraph), that "to establish the existence of a state of cellular disorder that affects all transcription profiles, the first step requires the choice of only one expression profile that could serve as a model for all profiles in a complex system."

#### Author response

*It has not been my intention to suggest that the use of only one profile would be sufficient to establish the existence of a state of disorder in a tissue (the "one fits all assumption"). This was also discussed in the text. To avoid such a misinterpretation the remark referenced here has been improved*.

4) There are numerous statements throughout the manuscript which are unsubstantiated and I cannot not agree with. For instance, the statement in the 'Results and Discussion' (section: Heritability of variant profiles): "The expression profiles of the two prostates clearly differ from each other, which suggests that a specific expression profile for the prostate does not exist and that these two profiles are disorderly. The similarity in disorderly expression profiles of a normal tissue and its tumor suggests the possibility of the disorderly condition in the normal tissue being a predisposing factor in the eventual appearance of the tumor."

#### Author response

*After a better separation of findings and speculations I assume that it is evident that the numerous unsubstantiated sayings throughout the manuscript are of a speculative nature*.

5) On several occasions the author suggests epigenetic changes to be responsible for 'disorderly' profiles. Yet, no supporting evidence is provided.

#### Author response

*"Epigenetic changes". The subheading 'Disorder in transcription can occur in euploid cells" has been changed into "Epigenetic origin of excessive variation in transcription". In this section three arguments were already given as supportive evidence for an epigenetic origin of the changes in expression profiles*.

6) The final 5-point conclusion/outlook in 'Results and Discussion' is pure speculation.

#### Author response

*The 5-point reflection should indeed be read as speculation. The subheading of this section has been altered according to this*.

7) Details of the additional 80 libraries mentioned in 'Methods' (section: Gene expression) should be included in Table 3.

#### Author response

*Details of the additional 80 libraries have been included in *table 3.

For the reasons outlined above, I find the manuscript not acceptable as Research or Review article and even questionable as Commentary/Hypothesis article. I declare that I have no competing interests.

#### Author response

*for the reasons outlined above and after the improvements made we trust that the manuscript is a valuable contribution. In our view this is clearly a research article*.
